# Alternative splicing-based therapeutics for neurodegenerative diseases: a dual-database bibliometric and NLP-driven analysis (2000–2025)

**DOI:** 10.3389/fmed.2026.1849726

**Published:** 2026-06-16

**Authors:** De-Zhu Liu, Gao-Lu Cheng, Chen-Feng Hu, Ru-Yang Li, Cong-Chu Yang, Ning-Chuan Chen, Xiang Li, De-You Jiang, Jia-Yi Chang

**Affiliations:** 1 Graduate School, Heilongjiang University of Chinese Medicine, Harbin, Heilongjiang, China; 2 Second Clinical Medical College, Heilongjiang University of Chinese Medicine, Harbin, Heilongjiang, China; 3 First Clinical Medical College, Heilongjiang University of Chinese Medicine, Harbin, Heilongjiang, China; 4 Department of Basic Medical Science, Heilongjiang University of Chinese Medicine, Harbin, Heilongjiang, China

**Keywords:** alternative splicing, antisense oligonucleotides, bibliometric analysis, clinical translation, natural language processing, neurodegenerative diseases

## Abstract

**Background:**

Neurodegenerative diseases (NDDs) are driven by complex molecular dysregulation, among which aberrant alternative splicing (AS) has emerged as a critical pathogenic mechanism. Despite the rapid development of splicing-targeted therapeutics, including antisense oligonucleotides (ASOs) and small molecules, comprehensive big-data syntheses mapping this translational landscape remain scarce. This study systematically analyzes the global research trends, conceptual frameworks, and clinical evolution of AS-based therapeutics for NDDs using an integrated bibliometric and natural language processing (NLP) approach.

**Methods:**

A dual-database retrieval strategy utilized the Web of Science Core Collection (WoSCC) and PubMed. The analysis targeted literature published between January 1, 2000, and December 31, 2025. A primary dataset of 620 records was extracted from WoSCC for comprehensive bibliometric mapping and Latent Dirichlet Allocation (LDA) topic modeling. A parallel analysis incorporated 10 targeted clinical trials and randomized controlled trials (RCTs) from PubMed using CLARA clustering to characterize high-evidence research. Bibliometric analyses, encompassing network topologies, citation bursts, and keyword evolution, were visualized using VOSviewer, CiteSpace, SCImago, and R.

**Results:**

Publication output exhibited sustained linear growth from 2000 to 2025. The United States and Western Europe emerged as the dominant collaborative hubs, while China exhibited high productivity but limited international integration. LDA topic modeling identified three core conceptual axes: molecular mechanisms, disease-specific pathological models (e.g., ALS, Alzheimer’s, and SMA), and translational methodological frameworks. Keyword trajectories delineated a transition from fundamental *in vitro* exploration to *in vivo* models, culminating in clinical “drug discovery” and “RNA” therapeutics. CLARA clustering of clinical trials demonstrated a stark concentration of splicing-modifying interventions in pediatric spinal muscular atrophy (SMA), revealing a dual-track paradigm of supportive care and molecular interventions.

**Conclusion:**

This multi-database bibliometric and NLP-driven study delineates the structural landscape of AS-based therapeutics for NDDs. It identifies a definitive paradigm shift from descriptive molecular biology to clinically actionable splicing interventions. These insights highlight the necessity to expand targeted RNA platforms beyond SMA into adult neurodegenerative populations, providing a strategic roadmap for future translational research.

## 1 Introduction

Neurodegenerative diseases (NDDs), including Alzheimer’s disease (AD), Parkinson’s disease (PD), and amyotrophic lateral sclerosis (ALS) (1), are pathologically defined by misfolded protein aggregates, progressive neuronal loss, and synaptic dysfunction, culminating in severe cognitive, motor, and behavioral decline (2). As a major subset of nervous system disorders—now the leading cause of disability-adjusted life years (DALYs) worldwide—NDDs contribute disproportionately to this global health crisis. Recent estimates indicate that nervous system disorders affect approximately 3.40 billion individuals, representing 43.1% of the global population, with a burden that is particularly acute in aging populations (3). In China, the 2025 China Alzheimer Report documented nearly 17 million cases of AD and related dementias (ADRD) in 2021, accounting for roughly 30% of the global total, with an age-standardized prevalence rate of 900.8 per 100,000—far exceeding the global average of 696.0 per 100,000. Notably, between 1990 and 2021, both the incidence and mortality of ADRD in China have risen sharply, with incidence increasing by over 240% and mortality by over 239% (4). Despite decades of intensive research, clinical management for NDDs remains largely symptomatic, and truly disease-modifying therapies are scarce. This therapeutic void carries a staggering socioeconomic cost (5). Globally, the number of people with dementia is projected to rise from 57.4 million in 2019 to 152.8 million by 2050 (6); in the United States alone, the annual indirect societal cost of Alzheimer’s disease—including unpaid caregiving and productivity loss—has been estimated at 832 billion (7). In China, ADRD contributed to over 10 million DALYs in 2021, with premature death (years of life lost, YLLs) accounting for 65.7% of this burden—a stark indicator of its devastating toll on patients, families, and healthcare systems.

Critically, the success of splicing-targeted interventions in pediatric disorders, such as spinal muscular atrophy (SMA), has thrown into sharp relief the lack of equivalent options for adult-onset NDDs. The central obstacle to developing disease-modifying NDD therapies is the extraordinary complexity of their pathogenesis, which involves a tangled web of genetic susceptibility, environmental triggers, and molecular dysregulation (8). Among the myriad disrupted molecular processes, alternative splicing (AS)—a fundamental post-transcriptional mechanism that generates proteomic diversity from a single gene—has emerged as a critical pathogenic driver (9). Under physiological conditions, AS precisely orchestrates the expression of proteins essential for neuronal development, survival, and function (10). However, mounting evidence confirms that aberrant AS is a conserved hallmark across multiple NDDs. Dysregulated splicing events alter the structure and function of key proteins, directly promoting toxic aggregate formation, disrupting cellular homeostasis, and accelerating neuronal death (11).

Consequently, correcting aberrant AS has become an increasingly attractive therapeutic strategy. Preclinical studies have shown that splicing-modulating agents, including antisense oligonucleotides (ASOs), small-molecule regulators, and CRISPR-based tools, can restore normal splicing patterns, reduce toxic protein accumulation, and preserve neuronal function in NDD models (12). Notably, ASO-based therapies targeting splicing defects have advanced to clinical trials for SMA and familial ALS, signaling a pivotal shift from basic discovery toward translational application (13).

Despite the rapid expansion of research on AS-based therapeutics for NDDs, the field currently lacks a systematic, data-driven synthesis of global trends, key collaborative networks, and emerging frontiers (14). Traditional narrative reviews, while valuable, typically focus on specific mechanisms or diseases, failing to capture cross-disciplinary connections, regional disparities, or the evolution of thematic patterns (15). This methodological gap calls for an integrative approach.

Here, we combine bibliometric analysis with Natural Language Processing (NLP), specifically Latent Dirichlet Allocation (LDA) topic modeling. This dual approach allows us to systematically quantify publication dynamics, identify core research clusters, and extract latent semantic themes from a large corpus of unstructured text (16). To our knowledge, this study represents the first comprehensive bibliometric and NLP-driven analysis of global AS-based therapeutic research for NDDs spanning 26 years (2000–2025). By mapping research hotspots, collaborative structures, and technological evolution, we aim to deliver a strategic roadmap—for researchers, clinicians, and policymakers—to foster interdisciplinary cooperation and accelerate the translation of splicing-targeted therapies from bench to bedside. To conduct an objective, data-driven analysis of the field’s semantic architecture, this study combines natural NLP with unsupervised machine learning. NLP transforms unstructured textual data (titles and abstracts) into computable features, enabling quantitative analysis of latent linguistic patterns. LDA is a generative probabilistic topic model that posits each document as a mixture of latent topics, and each topic as a distribution over words; it has been extensively utilized to uncover hidden thematic structures in large scientific corpora. Complementing LDA, Clustering for Large Applications (CLARA) is a k-medoids partitioning algorithm optimized for processing large datasets, offering robust clustering of heterogeneous clinical trial records without being sensitive to outliers. By applying these techniques to a dual-database corpus covering the period from 2000 to 2025, we aim to objectively define core research axes, track thematic shifts, and characterize the clinical trial landscape beyond traditional bibliometric indicators.

## 2 Materials and method

### 2.1 Eligibility criteria and screening procedures

A dual-database retrieval framework was employed to ensure robust bibliometric mapping and granular clinical coverage. The Science Citation Index Expanded (SCI-E) within the WoSCC served as the primary data source, selected for its standardized citation architecture essential for high-fidelity network analysis. Complementarily, PubMed was queried to conduct a dedicated analysis of clinical trials and randomized controlled trials (RCTs); these evidence types are critical for assessing translational impact but are often underrepresented in multidisciplinary citation indices (17). Conversely, the SCI-E dataset was chosen for its standardized citation indexing, which is essential for co-citation and burst detection analyses not supported by PubMed.

We systematically searched the WoSCC and PubMed. The search strategy combined keywords from three domains: alternative splicing, therapeutic interventions, and neurodegenerative diseases. For the SCI-E dataset, we applied the query to the Topic (TS) field. For PubMed, we combined Title/Abstract keywords with Medical Subject Headings (MeSH). To focus on clinical evidence, we restricted the PubMed results to records indexed as “Clinical Trial” or “Randomized Controlled Trial.” The complete search strings for both databases are detailed in Supplementary Table 1.

**FIGURE 1:**
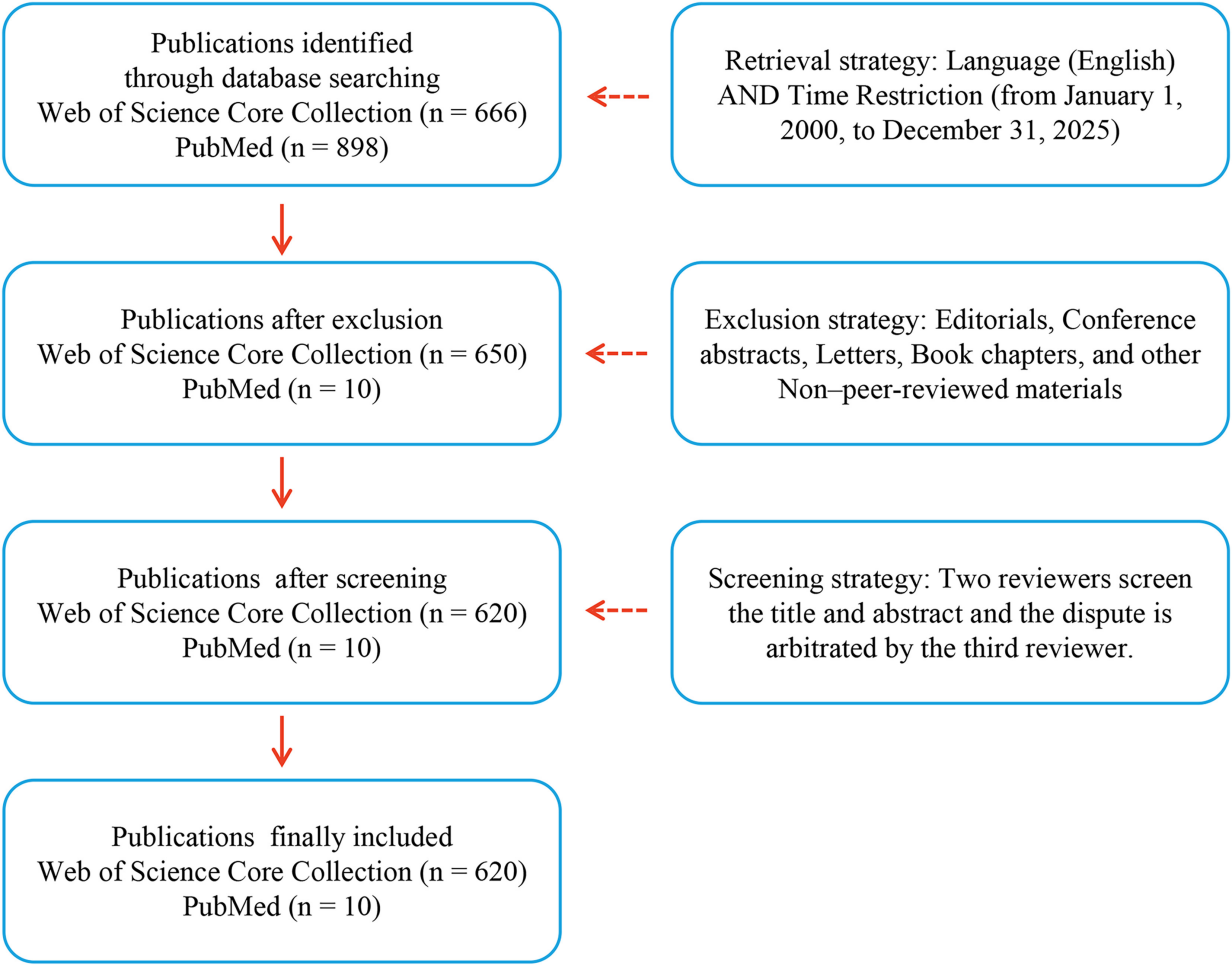
PRISMA flow diagram of the literature search and selection process.

Figure 1 illustrates the study selection workflow. The retrieval window extended from January 1, 2000, to December 31, 2025. To ensure linguistic consistency and uphold peer-review standards, only English-language articles and reviews were included in the SCI-E dataset. PubMed was searched independently to identify clinical trials and randomized controlled trials, restricted to English-language records. Editorials, conference abstracts, letters, book chapters, and other non-peer-reviewed materials were excluded from both sources. After data acquisition, all records underwent a standardized quality-control process. Duplicate entries were removed, and two reviewers independently screened titles and abstracts to eliminate studies unrelated to splicing-focused neurodegenerative therapeutics, such as research limited to oncology or non-neurological disorders. A third reviewer resolved any discrepancies. This multistep screening process ensured the accuracy of the final dataset and supported the reproducibility of subsequent bibliometric and thematic analyses. The final analysis included 620 articles from SCI-E dataset and 10 from PubMed.

The significant difference in sample size between SCI-E dataset (*n* = 620) and PubMed (*n* = 10) is not a methodological shortcoming but an accurate reflection of the field’s clinical maturity. The SCI-E dataset mainly consists of basic research, animal models, mechanistic studies, and non-interventional observations. In contrast, the PubMed search was intentionally limited to high-evidence study types (“Clinical Trial” or “Randomized Controlled Trial”). Under this strict filter, only 10 articles met the criteria. This scarcity emphasizes a key finding of our study: high-evidence interventional research on splicing-based therapeutics is still in its infancy, with a marked concentration in SMA and minimal presence in adult neurodegenerative diseases apart from SMA.

### 2.2 Bibliometric analysis

Bibliometric analyses were performed using Microsoft Excel 2024, Scimago Graphica Beta 1.0.53, CiteSpace 6.3 R3, VOSviewer 1.6.20, and the R package Bibliometrix 5.2.0. Microsoft Excel was used for preliminary data cleaning and tabulation. Scimago Graphica, a multidimensional scientific visualization platform, was applied to generate geographic maps, institutional collaboration chord diagrams, and optimized network layouts. CiteSpace and VOSviewer were used to conduct knowledge-structure mining and network visualization, including co-authorship networks, co-citation networks, keyword co-occurrence maps, burst detection analyses, and dual-map overlays to reveal disciplinary linkages and research evolution. Bibliometrix provided advanced statistical profiling and thematic analysis, offering deeper insights into research fronts, conceptual structures, and temporal trends (18).

### 2.3 Natural language processing and topic clustering

NLP techniques were used to construct and analyze the primary text corpus derived from article titles and abstracts, allowing systematic characterization of its thematic structure (19). The corpus was standardized through stop word removal, term-frequency filtering, and elimination of domain-specific noise to ensure reproducible and robust results. Two unsupervised learning methods were implemented in R. LDA, a probabilistic topic model that identifies latent semantic structures by estimating word–topic and topic–document distributions, was applied to extract the dominant themes in the SCI-E dataset. CLARA, a k-medoids partitioning algorithm optimized for large datasets, was used to classify clinical trial records retrieved from PubMed (20). Thematic prominence, topic structures, and cluster compositions were visualized using ggwordcloud and factoextra.

Although CLARA clustering is generally designed for moderate-to-large datasets, it remains stable and interpretable even with as few as 10 high-dimensional text vectors, particularly when the semantic structure is clear. The title/abstract corpus of these 10 trials was sufficient to uncover two core clusters—SMA-targeted molecular interventions versus general methodological descriptors in adult NDDs—and to further clarify the dual-track SMA landscape (supportive care versus molecular therapy). Incorporating non-RCT or lower-evidence records into the analysis to increase the sample size would introduce methodological heterogeneity and reduce the interpretability of the clinical clustering results.

Supplementary Table 2 lists the software used for data analysis and visualization.

## 3 Results

### 3.1 Literature overview and annual publication trends

**FIGURE 2:**
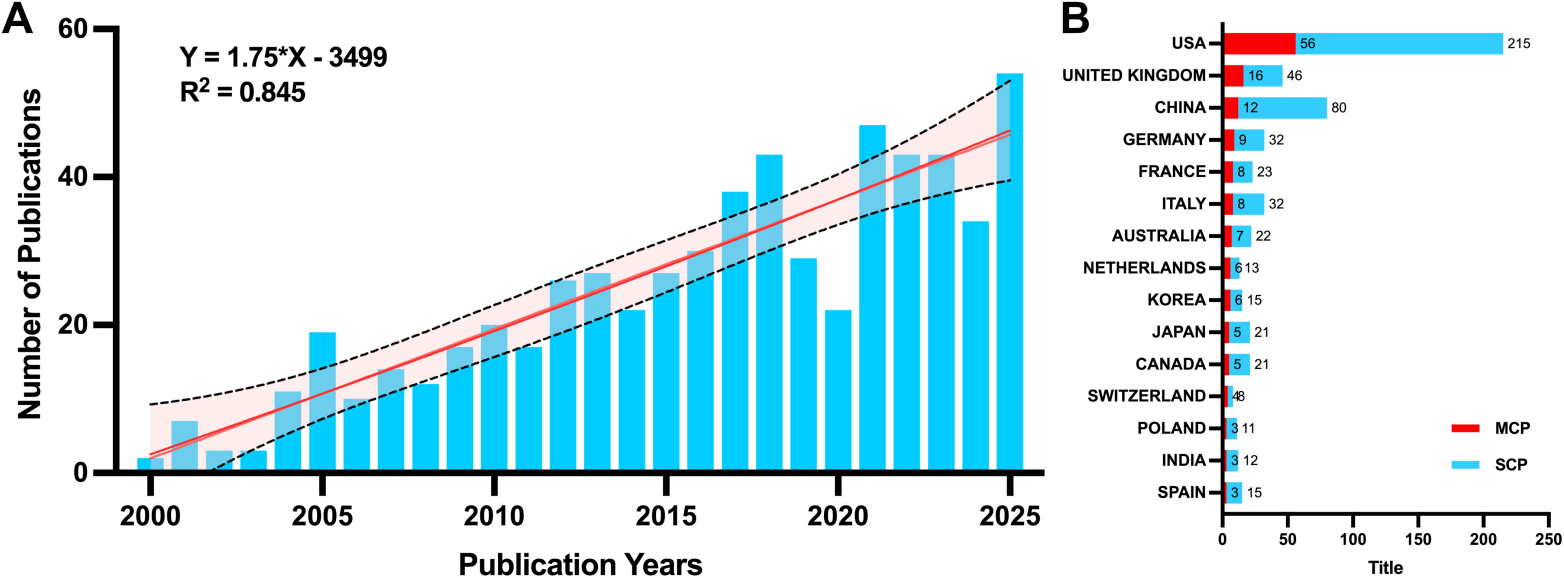
Annual publication output and corresponding author collaboration patterns (2000–2025). **(A)** Annual number of publications on alternative splicing–based therapeutics for neurodegenerative diseases from 2000 to 2025. **(B)** Distribution of corresponding-author publications by country, stratified into Single-Country Publications (SCP, dark blue) and Multiple-Country Publications (MCP, light blue).

As shown in Figure 2A, the annual number of publications in the field of alternative splicing-based therapeutics for neurodegenerative diseases exhibited a steady increase over the 26-year period, rising from 10 in 2006 to 54 in 2025. A temporary decline was observed in 2019–2020, which coincided with the global COVID-19 pandemic, a period associated with widespread disruptions in research activities across many biomedical fields. Linear regression analysis produced a fitted equation of Y = 1.75X − 3499 (where Y represents the annual number of articles and X represents the year), with a coefficient of determination of *R*^2^ = 0.845 (*p* < 0.001), indicating that the linear model accounts for 84.5% of the variance in publication output over time. This corresponds to an average annual increase of approximately 1.75 publications, which reflects the growing research interest in this domain.

As shown in Figure 2B, publications were categorized based on the country of the corresponding author. Single-country publications (SCP) were defined as articles in which all authors were affiliated with institutions in the same country as the corresponding author. Multiple-country publications (MCP) refer to articles with at least one co-author affiliated with an institution in a different country from that of the corresponding author. The MCP ratio (MCP%) was calculated as the proportion of MCP among total publications for each country. The United States contributed the largest number of corresponding author articles (215, 34.7%), with 159 SCP and 56 MCP, yielding an MCP ratio of 26.0%. This represents a moderate level of international collaboration, which is substantially lower than that of several European countries (e.g., UK 34.8%, France 34.8%). China ranked second in corresponding author articles (80, 12.9%) but exhibited the lowest MCP ratio (15.0%) among major contributors, with only 12 of its 80 articles involving international co-authors. European countries exhibited a distinctive pattern of high productivity coupled with a strong collaborative orientation. The United Kingdom (46 articles, MCP% = 34.8%), France (23 articles, MCP% = 34.8%), and Germany (32 articles, MCP% = 28.1%) all maintained MCP ratios above 28%.

### 3.2 Country and regional analysis

**FIGURE 3:**
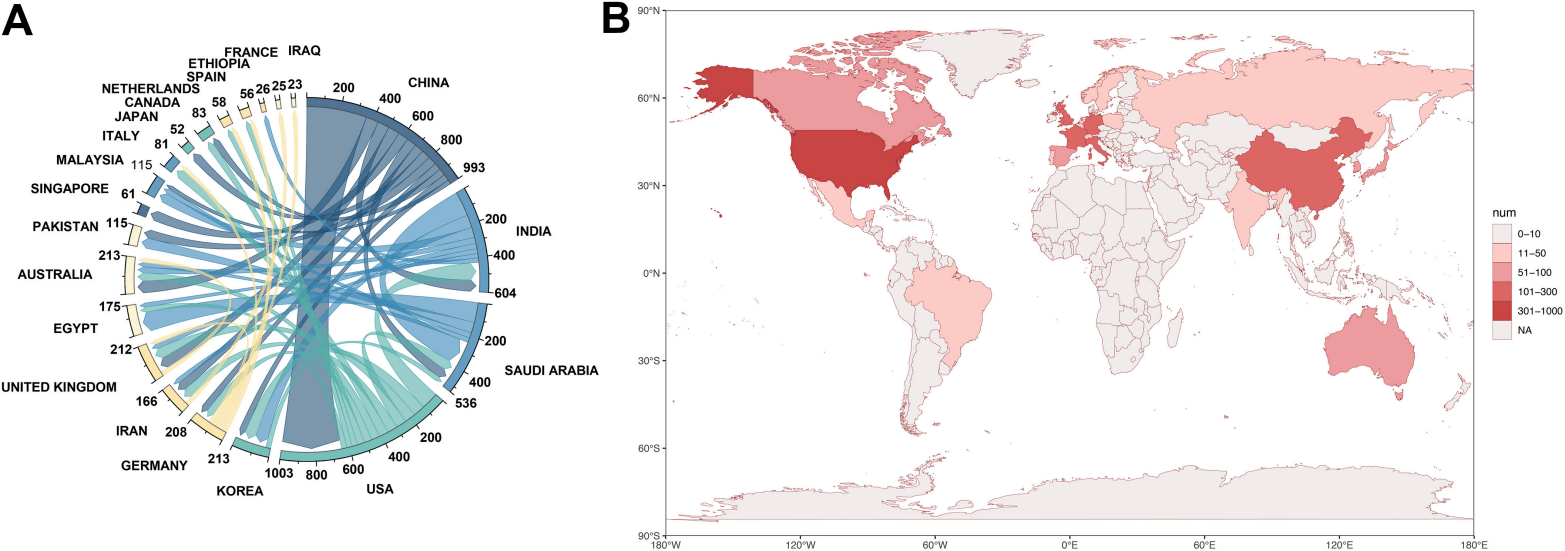
Geographic distribution and international collaboration networks. **(A)** World map showing country-level publication volume. **(B)** Chord diagram depicting collaborative relationships among countries.

Figure 3A maps the international collaborations. The network centers heavily on the United States. Key US partnerships include China (27 collaborations), the United Kingdom (22), Italy (16), France (14), Canada (13), and Germany (11). European nations form a tightly interconnected regional cluster, with notable intra-European links involving the UK, Italy, Germany, France, and Poland (5–8 collaborations each). In Asia, China and Japan drive moderate collaborative activity. Beyond its major US partnership, China shows limited international links (≤3 collaborations per country). Japan predominantly collaborates with the US (8), Germany (4), and the UK (4).

Figure 3B illustrates the geographic distribution of publications. The United States led the field with 875 publications. Notably, this output exceeded the combined total of the next three countries: China (293), the United Kingdom (152), and Germany (128). Italy (125) completed the top five. France, Japan, Canada, Spain, and Australia rounded out the top ten. Overall, research activity is heavily concentrated in North America and Western Europe, with China and Japan serving as the primary Asian contributors.

Table 1 details the top 10 countries by publication output, total citations (TC), centrality, and total link strength (TLS). The US dominates all metrics, ranking first in publications (261), TC (20,432), centrality (0.63), and TLS (173). China ranks second in publications (95). However, its TC (2,105), centrality (0.02), and TLS (57) lag significantly behind the US and major European countries. Conversely, Japan demonstrates high research impact despite moderate productivity. Ranking eighth with 32 publications, Japan achieved the highest mean citation rate (182.3 per paper; TC = 5,835), outperforming several higher-output nations.

**TABLE 1:** The top 10 countries by publication output and collaboration metrics.

Rank	Country	PN	TC	Centrality	TLS
1	United States	261	20,432	0.63	173
2	China	95	2,105	0.02	57
3	United Kingdom	63	6,418	0.24	82
4	Germany	51	4,657	0.24	47
5	Italy	47	5,190	0.20	67
6	France	46	4,703	0.24	60
7	Canada	38	3,483	0.01	43
8	Japan	32	5,835	0.11	33
9	Australia	26	1,230	0.01	32
10	Netherlands	24	794	0.03	30

PN, publication number; TC, total citations; TLS, total link strength.

### 3.3 Institutional analysis

**FIGURE 4:**
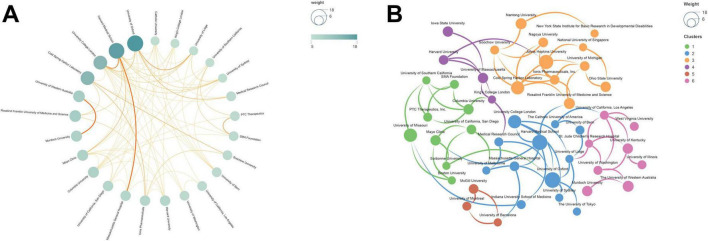
Institutional collaboration networks among institutions. **(A)** Chord diagram illustrating pairwise collaboration frequencies between institutions. **(B)** Network clustering of institutional collaborations.

Figures 4A,B map the collaborative network among the ten most productive institutions. The strongest partnership exists between Harvard Medical School and Massachusetts General Hospital, followed by the University of Western Australia and Murdoch University (Figure 4A). Overall, the network exhibits strong clustering: intra-cluster collaborations are dense, whereas inter-cluster connections remain limited (Figure 4B).

Table 2 details the top ten institutions. Harvard University leads in output (PN = 18; TC = 1,299; TLS = 22), followed by the University of London (PN = 17; TC = 856; TLS = 34) and INSERM (PN = 15; TC = 836; TLS = 6). US institutions account for half of the top ten, including Harvard Medical School (PN = 13) and Boston Children’s Hospital (PN = 8). France also features prominently, represented by INSERM, CNRS (PN = 9), and APHP (PN = 9). Notably, despite its lower publication count, Boston Children’s Hospital achieved the highest TC (2,196) and citations per publication (274.5).

**TABLE 2:** The top 10 institutions by publication output and collaboration metrics.

Rank	Institution	Country	PN	TC	TLS
1	Harvard University	USA	18	1,299	22
2	University of London	UK	17	856	34
3	Institut National de la Santé et de la Recherche Médicale (INSERM)	France	15	836	6
4	Harvard Medical School	USA	13	1,244	15
5	Harvard University Medical Affiliates	USA	13	1,133	23
6	University College London	UK	13	500	4
7	University of California System	USA	9	375	5
8	Centre National de la Recherche Scientifique (CNRS)	France	9	631	8
9	Assistance Publique–Hôpitaux de Paris (APHP)	France	9	290	7
10	Boston Children’s Hospital	USA	8	2,196	9

PN, publication number; TC, total citations; TLS, total link strength.

### 3.4 Analysis of author productivity and collaboration

**FIGURE 5:**
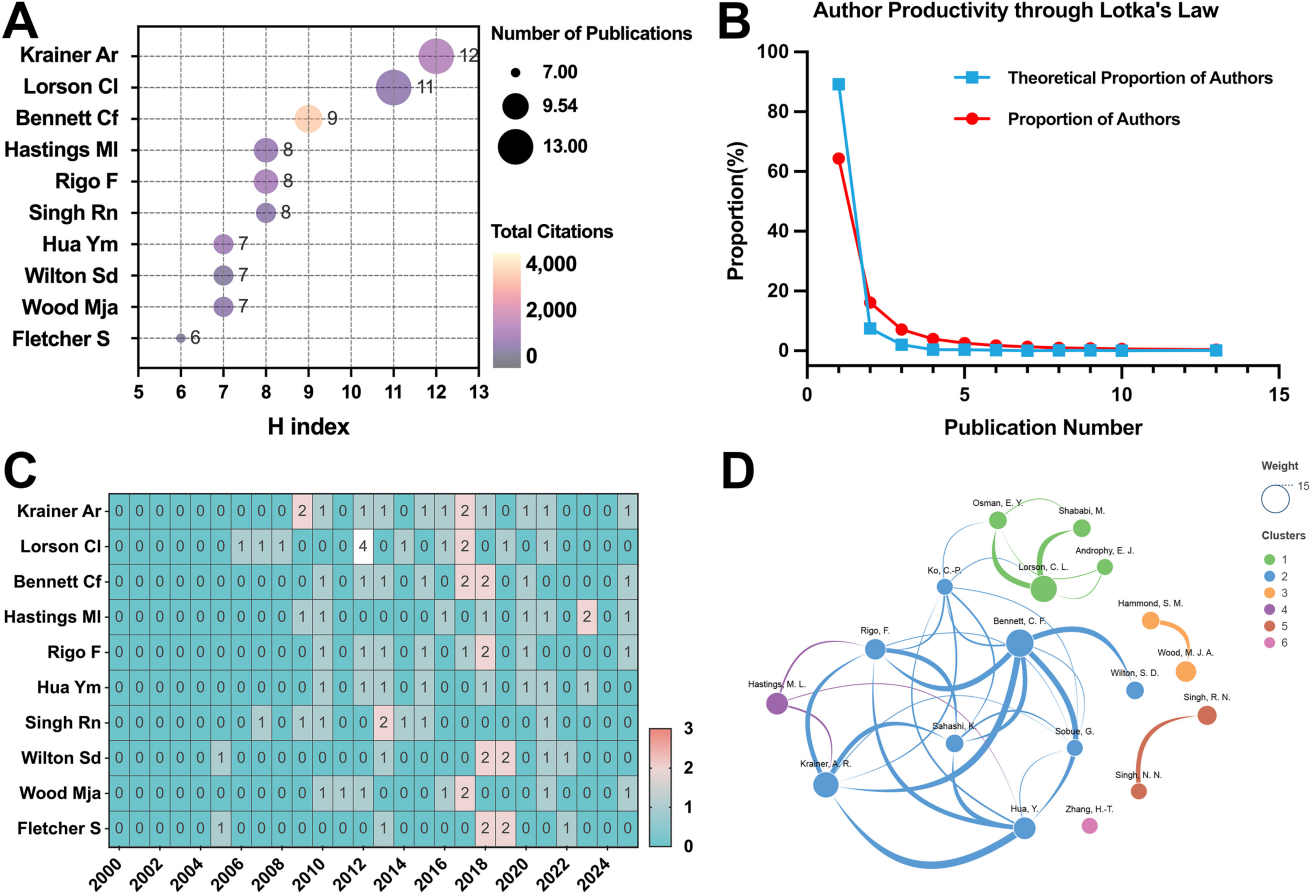
Author productivity, impact, collaboration networks, and temporal publication patterns. **(A)** Bubble chart correlating H-index, publication number (bubble size), and total citations (x-axis) for the ten most productive authors. **(B)** Observed author productivity distribution (red curve) versus Lotka’s law theoretical expectation (blue curve). **(C)** Temporal heatmap showing annual publication frequency per author (2000–2025). Color intensity ranges from blue (low) to pink (high). **(D)** Author collaboration network.

Figure 5A presents a bubble chart that depicts the relationship between the H-index, the total number of publications, and the TC for the ten most productive authors in the field, thus facilitating a multidimensional comparison of scholarly productivity and impact. The distribution of authors along the H-index axis is relatively concentrated, ranging from 7 to 13. Notably, Krainer A.R. (H-index = 13, PN = 12, TC = 1,244) and Lorson C.L. (H-index = 13, PN = 11, TC = 500) have the highest H-index, which reflects their long-term and consistent contributions to the field. However, their citation counts vary significantly, indicating that Krainer’s work has a substantially higher per-paper impact. A notable outlier is Bennett C.F. Despite publishing a moderate number of 9 papers and having an H-index of 10, he received the highest TC (3,637) among all authors. This pattern highlights a research profile that is characterized by a small number of highly influential papers, rather than high-volume production.

Figure 5B depicts the distribution of author productivity within the field, plotted in comparison to the theoretical distribution predicted by Lotka’s law. The x-axis indicates the number of documents written by each author, and the y-axis shows the proportion of authors who have contributed that number of papers. The red curve represents the observed proportion of authors, whereas the blue curve represents the theoretical values derived from the generalized Lotka’s law (with an exponent of approximately 2), which indicates that the number of authors publishing n papers is inversely proportional to n2. As shown in Figure 5B, the observed distribution shows a significant skew: a large majority of authors (89.2%) contributed only one paper, while the theoretical proportion for single-paper authors is 64.3%. On the contrary, for authors publishing two or more papers, the observed proportions are consistently lower than the theoretical expectations. For example, authors with two publications make up 7.5% of the total (theoretical: 16.1%), and those with three publications account for 2.0% (theoretical: 7.1%). This pattern indicates a more extreme concentration of low-productivity authors than predicted by Lotka’s law, along with a relative scarcity of moderately productive authors. Notably, the tail of the distribution (≥5 papers) shows that only a small fraction of authors (0.9%) published five or more papers. This finding indicates that a small group of highly productive researchers generates a significant portion of the field’s publications.

Figure 5C depicts a temporal heatmap that shows the annual publication frequency of the ten most productive authors in the field from 2000 to 2025. Each cell indicates the number of publications written by an individual in a specific year, and the color intensity varies from blue (low output) to pink (high output). Before 2010, publications were few and mainly limited to a few authors like Lorson C.L. and Singh R.N., indicating the early stage of the field. Around 2012 and later, a wider range of authors started contributing more regularly. Notably, Bennett C.F., Wilton Sd and Rigo F. showed continuous productivity from the mid-2010s to 2025, with multiple years of high-output activity (around 2 papers/year), highlighting their continuous leadership in the development of splicing-targeted therapy.

Table 3 summarizes the 10 most productive authors in the field, ranked by publication output (PN). Geographically, the United States dominates the list, with eight of the top 10 authors, followed by the United Kingdom (Wood, M.J.A.) and Japan (Sahashi, K.). This concentration highlights the leading role of U.S.-based research groups in the field. Notably, a dissociation between publication output and citation impact is evident. Krainer A.R. (PN = 12, TC = 1,073) and Bennett C.F. (PN = 8, TC = 936) have exceptionally high citation counts relative to their publication output. In contrast, Lorson C.L., the most prolific author (PN = 13), has a comparatively moderate total number of citations (TC = 500). As illustrated in Figure 5D, TLS further distinguishes the authors. Krainer (30), Hua (28), Bennett (27), Rigo (26), and Sahashi (24) exhibit high TLS, indicating their integration into dense collaborative networks. Notably, Sahashi K., the sole Asian author in the top 10, attains a high TLS (24). Conversely, authors like Hastings M.L. (5) and Wood M.J.A. (5) have lower TLS values, suggesting more localized or independent research endeavors.

**TABLE 3:** The top 10 authors by publication output and collaboration metrics.

Rank	Author	Country	PN	TC	TLS
1	Lorson, Christian L.	USA	13	500	14
2	Krainer, Adrian R.	USA	12	1,073	30
3	Hua, Yimin	USA	9	904	28
4	Hastings, Michelle L.	USA	9	631	5
5	Bennett, C. Frank	USA	8	936	27
6	Rigo, Frank	USA	8	909	26
7	Wood, Matthew J. A.	UK	8	465	5
8	Singh, Ravindra N.	USA	7	391	5
9	Sahashi, Kentaro	Japan	6	753	24
10	Osman, Erkan Y.	USA	6	232	9

PN, publication number; TC, total citations; TLS, total link strength.

### 3.5 Journal analysis

**FIGURE 6:**
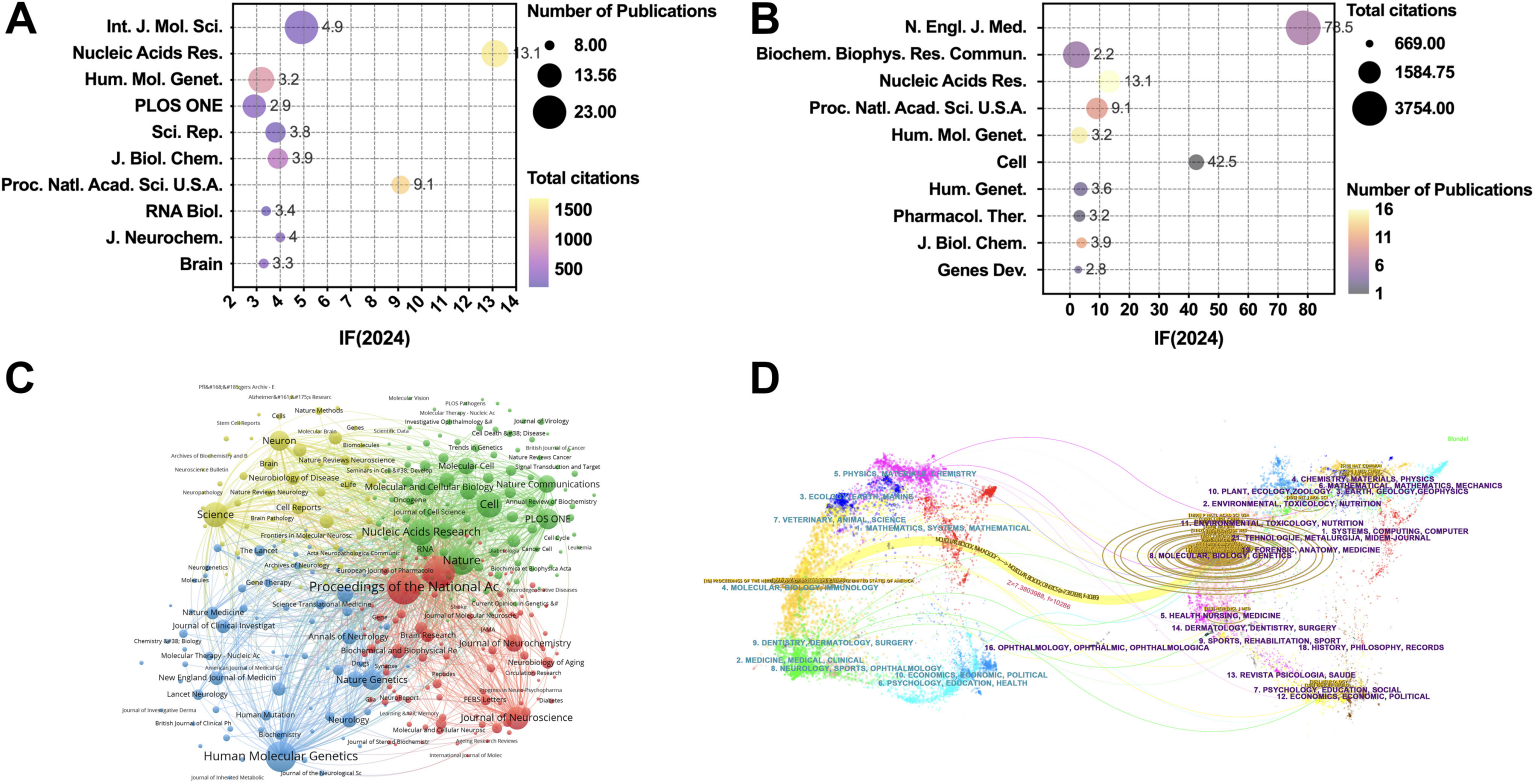
Journal productivity, citation impact, co-citation networks, and disciplinary mapping. **(A)** Bubble chart of the ten most productive journals. **(B)** Bubble chart of the ten most cited journals. **(C)** Journal co-citation network. **(D)** Dual-map overlay of journals.

Table 4 details the metrics for the leading journals. Figures 6A,B map their Impact Factor (IF 2024), TC, and publication output. *Nucleic Acids Research* (*NAR*) stands out as a core journal. It combines high output (*n* = 16), a strong IF (13.1), and substantial citations (1,606). In contrast, the *International Journal of Molecular Sciences* leads in volume (*n* = 23). However, it yields lower citations (345) and a moderate IF (4.9). Several journals demonstrate massive impact with few publications. The *New England Journal of Medicine* published only six articles. Yet, it dominates TC (3,754) and IF (78.5). This underscores its clinical authority. *Cell* similarly generated 945 citations from a single publication. *Proceedings of the National Academy of Sciences* (*PNAS*) achieved 1,477 citations from just 10 articles. The co-citation network (Figure 6C) identifies *PNAS* as the central hub (citations = 1,891; TLS = 183,456). The top five co-cited journals form a tightly interconnected cluster. These include *PNAS*, *Journal of Biological Chemistry* (*JBC*), *Nature*, *Human Molecular Genetics*, and *NAR*. This clustering reflects robust integration across molecular biology and genetics. Finally, a dual-map overlay traces the thematic distribution (Figure 6D). A distinct yellow path links immunology and genetics. Specifically, genetics journals frequently cite research from immunology journals (*z* = 7.38, *f* = 10,286).

**TABLE 4:** The top 10 journals by publication output and impact metrics.

Rank	Journal	Country	JCR	IF (2024)	PN	TC	TLS
1	International Journal of Molecular Sciences	Switzerland	Q1	4.9	23	345	1,730
2	Nucleic Acids Research	United Kingdom	Q1	13.1	16	1,606	3,419
3	Human Molecular Genetics	United Kingdom	Q2	3.2	15	1,007	2,660
4	PLOS ONE	United States	Q2	2.6	13	388	1,285
5	Journal of Biological Chemistry	United States	Q2	3.9	11	709	593
6	Scientific Reports	United Kingdom	Q1	3.9	11	302	1,581
7	Proceedings of the National Academy of Sciences of the United States of America (PNAS)	United States	Q1	9.1	10	1,477	1,609
8	Brain	United Kingdom	Q1	11.7	8	405	697
9	Journal of Neurochemistry	United Kingdom	Q2	4	8	366	687
10	RNA Biology	United Kingdom	Q2	3.4	8	260	2,063

JCR, Journal Citation Reports; IF, impact factor; PN, publication number; TC, total citations; TLS, total link strength.

### 3.5 Keyword analysis

**FIGURE 7:**
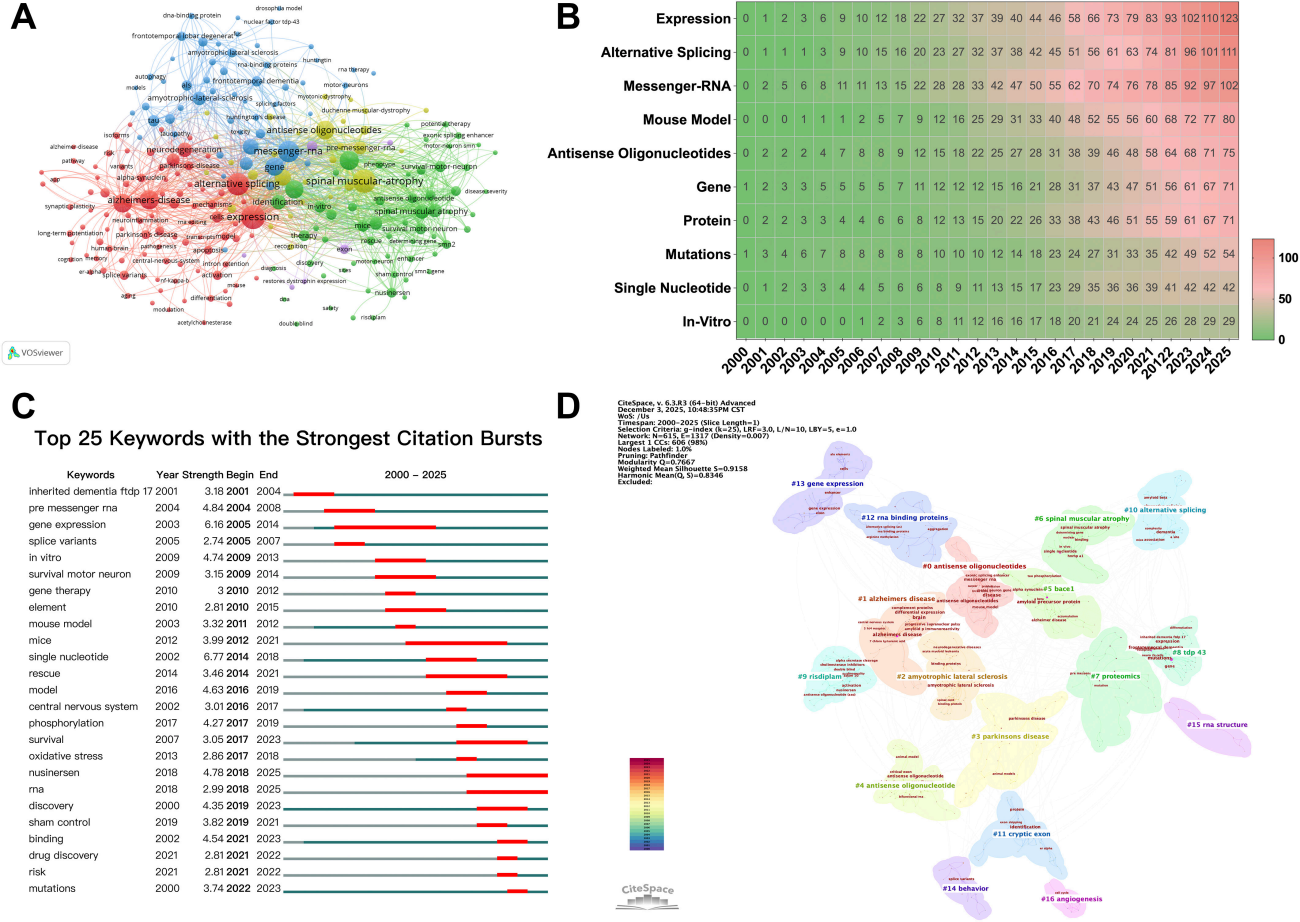
Keyword co-occurrence, temporal trends, citation bursts, and clustering analysis. **(A)** Keyword co-occurrence network. **(B)** Temporal heatmap of the ten most representative keywords (2000–2025). **(C)** Top 25 keywords with the strongest citation bursts (red bars). **(D)** Keyword clustering map (*Q* = 0.7667, *S* = 0.9158).

Table 5 details the specific keyword frequencies. Figure 7A visualizes the corresponding network. The five most frequent keywords are “expression” (*n* = 123), “spinal muscular atrophy” (*n* = 120), “alternative splicing” (*n* = 111), “messenger-RNA” (*n* = 102), and “Alzheimer’s disease” (*n* = 82). These terms form a densely interconnected core cluster. They dominate the entire network.

**TABLE 5:** The top 15 keywords by occurrence frequency and centrality.

Rank	Keywords	Frequency	Centrality
1	Spinal muscular atrophy	144	0.14
2	Alzheimer’s disease	139	0.44
3	Expression	119	0.14
4	Alternative splicing	111	0.19
5	Messenger RNA	102	0.18
6	Mouse model	79	0.13
7	Antisense oligonucleotides	73	0.15
8	Gene	69	0.1
9	Protein	69	0.1
10	Gene expression	59	0.12
11	Survival motor neuron	55	0.05
12	Mutations	48	0.08
13	Amyotrophic lateral sclerosis	47	0.11
14	Identification	44	0.12
15	Single nucleotide	36	0.02

A temporal heatmap tracks the annual frequencies of ten representative keywords from 2000 to 2025 (Figure 7B). Overall, research activity intensifies across most terms. This aligns with the field’s sustained growth. In the early phase (2000–2010), “expression,” “gene,” and “protein” dominated the literature. During the mid-phase (2011–2016), “mutations” and “single nucleotide” gained prominence. In the recent phase (2017–2025), “antisense oligonucleotides” show the most substantial increase. Concurrently, the “mouse model” maintains a consistently high frequency. The term “messenger-RNA” exhibits continuous linear growth throughout the entire period. This reflects its fundamental status in the field.

Figure 7C identifies the 25 keywords with the strongest citation bursts. This illustrates the phased evolution of research hotspots. In the early stage (2000–2010), “pre-mRNA” (strength = 4.84) and “gene expression” (strength = 6.16) predominated. During the middle stage (2010–2017), terms like “mice” (strength = 3.99) and “survival motor neuron” (strength = 3.15) emerged. The term “single nucleotide” (strength = 6.77) displayed the highest burst intensity overall (2014–2018). Recently (2018–2025), “drug discovery” (strength = 2.81) and “oxidative stress” (strength = 2.86) became primary focuses. Furthermore, the targeted therapeutic “nusinersen” (strength = 4.78) and the core entity “RNA” (strength = 2.99) remain in a burst status through 2025.

The co-occurrence network map is shown in Figure 7D. The network comprises 615 nodes and 1,317 edges. The network density is 0.007. Key metrics include a modularity value (Q) of 0.7667 and a weighted mean silhouette value (S) of 0.9158. The largest connected subgraph encompasses 98% of all nodes. These parameters indicate a highly significant clustering structure with excellent within-cluster homogeneity.

Spatially, “antisense oligonucleotides” (clusters #0 and #4) occupy the absolute core position. This technological hub connects upstream to fundamental molecular mechanisms. These include “alternative splicing” (#10), “cryptic exons” (#11), and “RNA-binding proteins” (#12). Downstream, it radiates to distinct disease-specific clusters. Examples include “Alzheimer’s disease” (#1), “amyotrophic lateral sclerosis” (#2), “Parkinson’s disease” (#3), and “spinal muscular atrophy” (#6). Key pathological targets like “BACE1” (#5) and “TDP-43” (#8) are also evident. A representative drug cluster for “risdiplam” (#9) appears in the network. Overall, this map delineates a clear knowledge trajectory. The field progresses from fundamental RNA mechanisms to clinical targeted therapy. Antisense oligonucleotide technology serves as the central bridge.

### 3.6 Latent Dirichlet Allocation topic modeling

**FIGURE 8:**
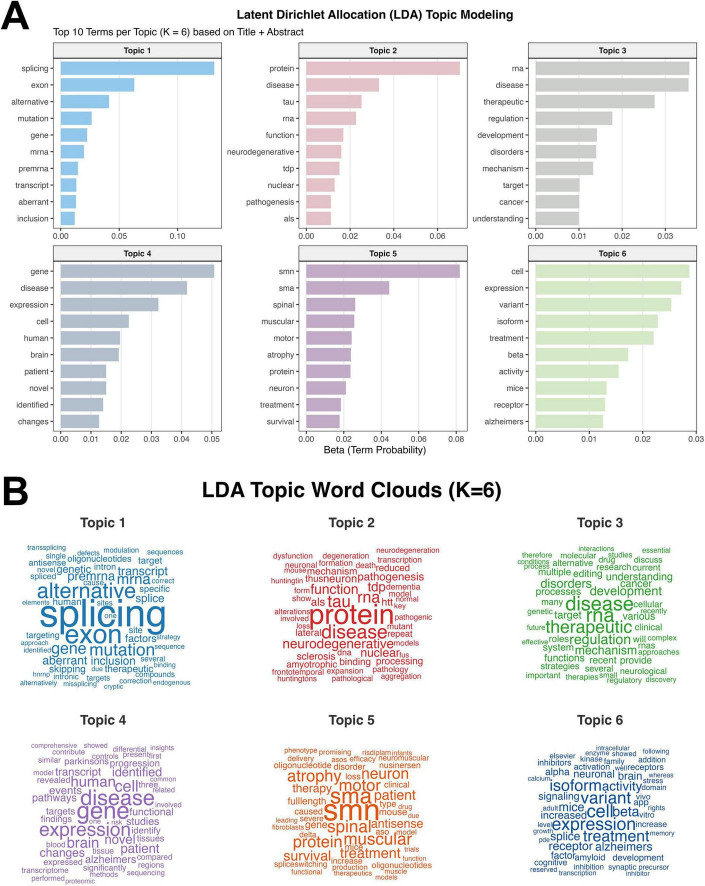
Latent Dirichlet Allocation (LDA) topic modeling analysis. **(A)** Top 10 terms for the six identified topics (*K* = 6) based on titles and abstracts. **(B)** Word clouds representing the core terms for each topic.

We employed LDA topic modeling on the SCI-E dataset to elucidate the latent thematic structure of research regarding alternative splicing in neurodegenerative therapeutics. Perplexity-based optimization identified a six-topic solution as the most robust model (Figure 8A). Topic labels were defined through expert curation of the ten highest-probability terms within each cluster, enabling a coherent interpretation of thematic domains. The analysis stratified the 620 included publications into three primary conceptual axes: molecular mechanisms, disease-specific pathological models, and translational methodological frameworks.

Within the molecular mechanism’s axis, Topic 1 delineates the fundamental regulatory architecture of splicing. Dominant terms such as splicing, exon, and mutation reflect intensive investigation into the cis- and trans-acting determinants of pre-mRNA processing and their critical role in maintaining transcriptomic fidelity. The disease-specific pathological axis comprises three mechanistically distinct clusters. Topic 2 characterizes RNA-binding retinopathies central to ALS and FTD, defined by high-probability terms including tau, TDP, and protein. Topic 6 highlights isoform-specific pathological signatures in Alzheimer’s disease, dominated by terms such as isoform, beta, and cell. Topic 5 encapsulates the therapeutic paradigm in SMA, anchored by clinically salient terms such as SMN, survival, and treatment, which underscore the translational success of splicing-modifying interventions. The translational methodologies axis bridges these mechanistic and disease-focused domains. Topic 3 aggregates terminology related to therapeutic strategy development (e.g., therapeutic, target, regulation), signaling the maturation of broad-spectrum RNA intervention platforms. Topic 4 encompasses transcriptomic profiling and biomarker discovery, with frequent terms such as gene, expression, human, and brain aligning with omics-driven approaches for identifying disease-relevant splicing signatures.

Complementing these quantitative distributions, word clouds were generated for each topic (Figure 8B). These visualizations illustrate the relative prevalence and weighting of constituent terms, providing an intuitive representation of the field’s underlying semantic structure.

### 3.7 CLARA clustering and temporal evolution of clinical trials

**FIGURE 9:**
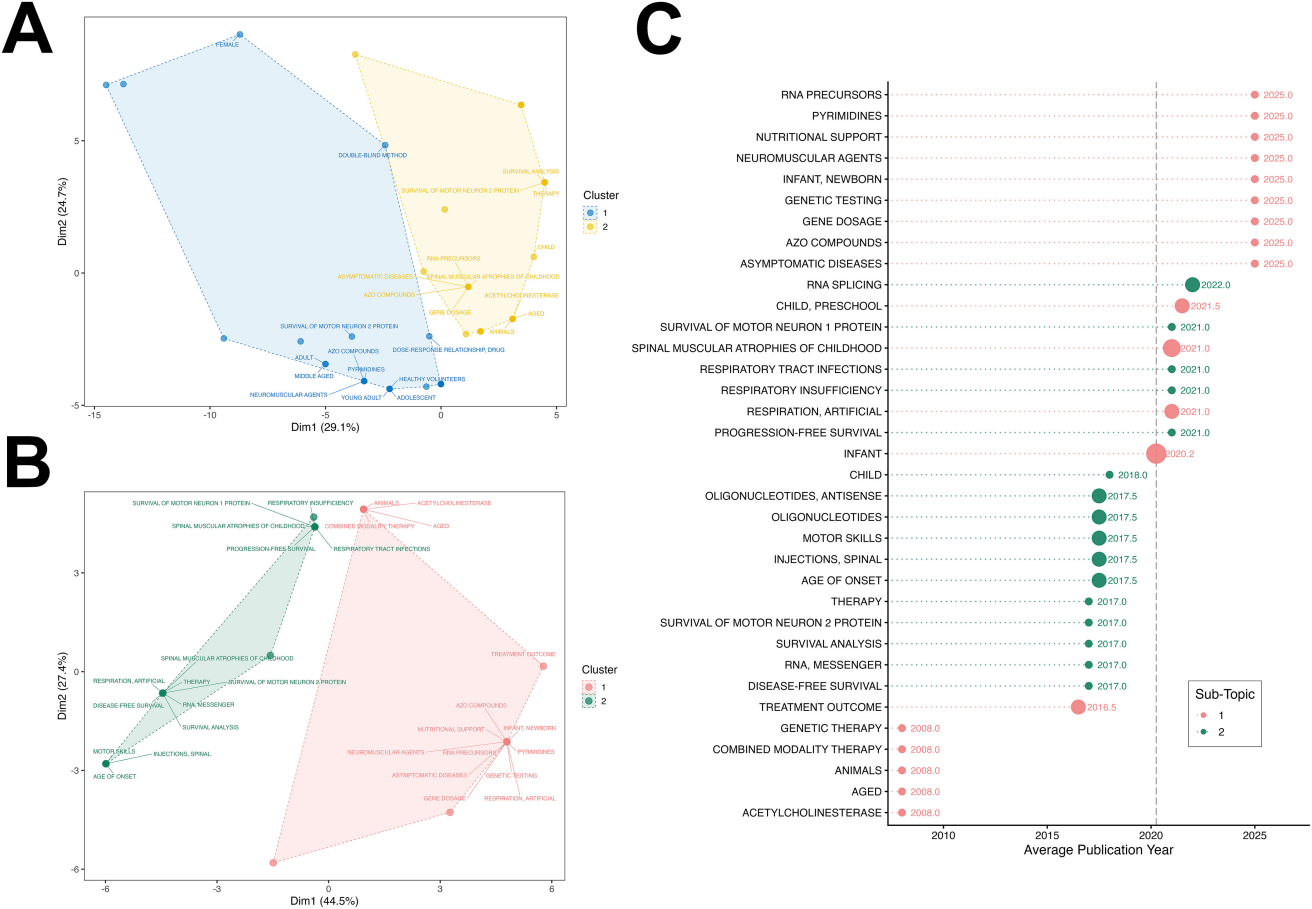
Clustering Large Applications (CLARA) analysis of the literature. **(A)** Initial clustering analysis of the extracted terms. **(B)** Secondary clustering analysis focusing on the major terms identified in the initial stage. **(C)** Temporal distribution of the topics derived from the secondary clustering analysis.

We applied CLARA clustering to PubMed clinical trials and RCTs. This maps the structural organization of high-evidence interventional research. Figure 9A visualizes this dimensionality-reduced space. Dimensions 1 and 2 explain 29.1 and 24.7% of the total variance, respectively. The subsampling procedure yielded highly stable partitioning. Two dominant clusters emerged. Cluster 1 (blue) features general methodological descriptors (“Double-Blind Method,” “Dose-Response Relationship”) and adult demographics (“Adult,” “Middle Aged”). It represents the broad infrastructure of neurodegenerative clinical trials. Cluster 2 (yellow) features pediatric and disease-specific terms (“Child,” “Spinal Muscular Atrophy,” “SMN2 Protein”). This indicates a stark concentration of splicing-modifying interventions in pediatric SMA cohorts over adult populations.

Figure 9B further resolves the SMA-focused structure. It reveals two distinct but co-existing domains. This functional split indicates a high maturity in SMA clinical research. Cluster 1 (green) represents the molecular intervention domain. It relies on mechanism-oriented terms, including “RNA, Messenger,” “Injections, Spinal,” and “SMN1/2 Protein.” Cluster 2 (pink) constitutes a clinical management framework. It is enriched with supportive-care terms like “Respiration, Artificial,” “Nutritional Support,” and “Combined Modality Therapy.”

Finally, temporal mapping traces a directional progression in therapeutic innovation (Figure 9C). The Supportive Era (pre-2016) features symptomatic and pre-clinical markers (“Acetylcholinesterase,” “Animals”). The ASO Breakthrough Era (2017–2020) shows a marked enrichment of core therapeutics (“Oligonucleotides, Antisense,” “Injections, Spinal”). The Precision and Screening Era (2021–2025) highlights early detection and prevention. It is dominated by terms like “Infant,” “ewborn,” “Genetic Testing,” and “Asymptomatic Diseases.”

### 3.8 Funding source dynamics across SMA clinical milestones

**FIGURE 10:**
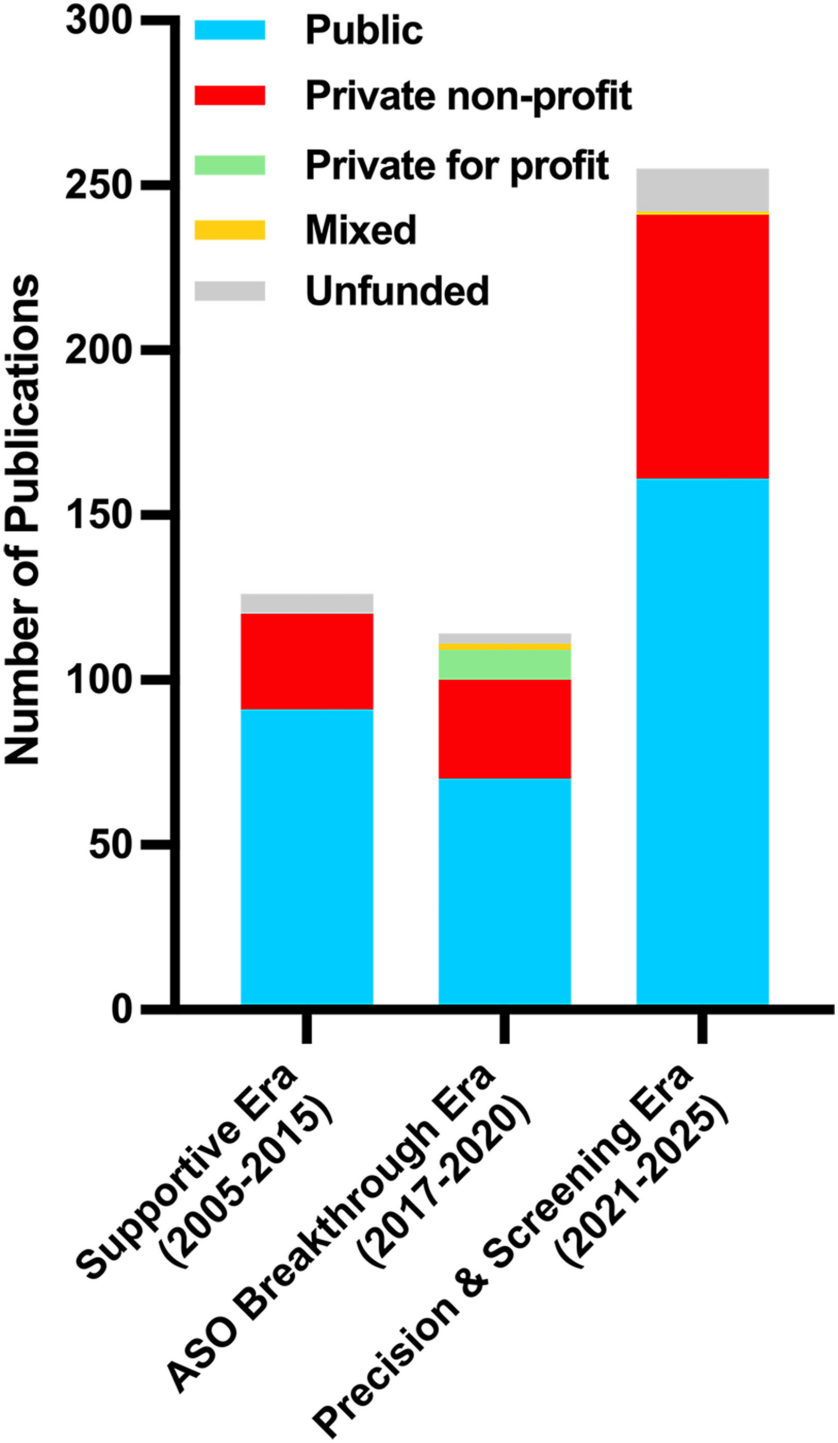
Evolution of funding patterns for spinal muscular atrophy (SMA) research across three clinical milestone eras.

To examine how funding patterns evolved in parallel with clinical advances in SMA—the most successful paradigm of splicing-targeted therapy—we aggregated annual funding data into three eras defined by the SMA clinical milestone framework (Figure 10): Supportive Era (2005–2015), ASO Breakthrough Era (2017–2020), and Precision and Screening Era (2021–2025). Funding sources were categorized as public, private non-profit, private for-profit, mixed, or unfunded. The distribution across eras is shown in Figure 10.

From the Supportive Era (*n* = 126) to the ASO Breakthrough Era (*n* = 114), public funding decreased from 72.2 to 61.4%, while private non-profit funding increased modestly from 23.0% to 26.3%. Private for-profit and mixed funding appeared for the first time in the ASO Breakthrough Era, accounting for 7.9 and 1.75% of publications, respectively. Unfunded publications comprised 4.8% in the Supportive Era and 2.6% in the ASO Breakthrough Era.

In the Precision Era (*n* = 255), public funding remained similar to the ASO Breakthrough Era (63.1%), whereas private non-profit funding rose further to 31.4%. Notably, private for-profit funding dropped to 0% during 2021–2025, with no for-profit-funded publications identified. Unfunded publications increased slightly to 5.1%.

Supplementary Table 3 lists the specific number of different types of funding in these three eras.

## 4 Discussion

### 4.1 Overview and publication trajectories

This study analyzed 620 publications from Web of Science and 10 from PubMed on alternative splicing-based therapeutics for neurodegenerative diseases. We used bibliometric methods to map the global research landscape. We assessed country and institutional output, author productivity, journal distribution, citation profiles, and keyword evolution. We also constructed a text corpus and applied NLP. These approaches allowed us to trace the historical development of the field, identify current research hotspots, and predict future research directions.

The annual publication trend from 2000 to 2025 (Figure 2A) reveals a sustained linear increase in publication output, with an average growth rate of 1.75 articles per year (*R*^2^ = 0.845). This steady expansion reflects the field’s maturation from foundational discoveries in splicing regulation to translational applications, paralleling milestones such as the FDA approval of nusinersen for SMA (2016) and the emergence of small-molecule splicing modifiers (e.g., risdiplam). The temporary decline from 2019 to 2020 coincides with the global slowdown in research during the COVID-19 pandemic, highlighting the vulnerability of scientific productivity to external disruptions. The subsequent rebound from 2021 to 2025 indicates a resilient and sustained interest in splicing-based therapeutics.

### 4.2 Global collaboration and structural disparities

As illustrated in Figure 2B, the United States, despite being the undisputed collaborative hub (highest centrality and TLS), exhibited a moderate MCP ratio (26.0%), lower than most European counterparts. This apparent paradox indicates that the US operates as a passive collaborative magnet rather than an active pursuer of international partnerships. Its scientific superiority, substantial funding, and world-leading infrastructure naturally draw foreign collaborators. This pattern has implications for global science: while it guarantees US leadership, it may also restrict the diversity of perspectives entering the US research system and diminish opportunities for mutual learning. China ranked second in publication output but exhibited the lowest MCP ratio among major contributors (15.0%), along with low centrality (0.02) and moderate citation impact. Several factors may contribute: language barriers, a domestically oriented research agenda, publication in lower-profile journals, and limited structural mechanisms for sustained international collaboration (21). This indicates that the inadequate integration into global research networks may limit the influence of Chinese scholarship in this field.

The clustering structure of the institutional collaboration network (Figure 4B) indicates that intra-cluster links are dense, whereas inter-cluster connections are limited. This pattern suggests that institutional collaboration in this field is primarily driven by academic affinity and institutional affiliations, with core institutions playing a pivotal role in organizing and facilitating collaborative networks.

The analysis of author productivity (Figure 5B) shows a significant deviation from the classical Lotka’s law, as 89.2% of authors contribute only one paper, which significantly surpasses the theoretical expectation of 64.3%. In contrast, the proportion of authors publishing two or more papers is consistently lower than the prediction, suggesting a shortage of moderately productive researchers. This skewed distribution is characterized by an extreme concentration of low-productivity authors and a small core of highly prolific contributors (0.9% of authors producing ≥5 papers). This phenomenon may reflect the high threshold for continued contribution in this field. Conducting impactful research in splicing-targeted therapeutics often requires access to advanced RNA technologies, disease models, and interdisciplinary collaborations—capabilities that are concentrated in a limited number of well-funded laboratories. As a result, many early-career researchers or those from resource-limited settings may publish one paper and then leave the field, unable to sustain a long-term research program (22). This also indicates the importance of supporting established research hubs.

The country, institutional, and authorship concentration observed in this study highlights the field’s dependence on a limited number of scientifically advanced regions and high-capacity research centers. Although this concentration has undoubtedly expedited methodological innovation and translational progress, it also exposes a structural imbalance (23). Expanding participation from underrepresented countries and institutions is crucial for diversifying research perspectives, enhancing knowledge exchange, and ultimately strengthening the global ability to develop alternative splicing–based therapeutics for neurodegenerative diseases.

### 4.3 Journal core and the volume-impact gap

Figures 6A,B encompass journals with diverse disciplinary scopes, spanning from broad multidisciplinary and general science journals to specialized journals focused on molecular biology, neuroscience, and RNA. The publication output rank (Figure 6A) is dominated by specialized molecular biology journals. The *International Journal of Molecular Sciences* (*IJMS*) leads in output (23 articles) but has a moderate citation impact (345 citations; IF 2024 = 4.9). In contrast, the total citation-based rank (Figure 6B) shows a more diverse landscape, including top-tier clinical journals (such as *The New England Journal of Medicine*, *NEJM*), method-focused journals (*Biochemical and Biophysical Research Communications*, *BBRC*), and high-impact multidisciplinary journals (*Proceedings of the National Academy of Sciences*, *PNAS*; *Cell*). Notably, only four journals appear on both lists (*Journal of Biological Chemistry*, *Human Molecular Genetics*, *PNAS*, *Nucleic Acids Research*), highlighting a functional difference between high-output and high-impact journals. Journals that dominate in terms of publication volume are not necessarily those that accumulate the highest citations.

### 4.4 Keyword dynamics and the translational shift

Figure 7C presents the top 25 keywords with the strongest citation bursts from 2000 to 2025, clearly illustrating the phased evolution of research hotspots in this field. In the early stage (2000–2010), keywords such as “pre-mRNA” (strength = 4.84) and “gene expression” (strength = 6.16, exhibiting an extremely high burst intensity) predominated, indicating that research during this period was primarily grounded in fundamental molecular mechanism exploration and in vitro experiments. In the middle stage (2010–2017), a cluster of keywords, including “mice” (strength = 3.99) and “survival motor neuron” (strength = 3.15), emerged as burst terms. Notably, “single nucleotide” (strength = 6.77) displayed the highest burst intensity across the entire spectrum (2014–2018). This shift signifies a transition of research focus from in vitro mechanisms to in vivo animal models, with a precise targeting of genetic loci associated with specific neurological diseases. In recent years (2018–2025), research hotspots have markedly pivoted toward clinical translation and application, with emerging foci such as “drug discovery” (strength = 2.81) and “oxidative stress” (strength = 2.86). Of particular note, the representative targeted therapeutic agent “nusinersen” (strength = 4.78) and the core molecular entity “RNA” (strength = 2.99) have maintained burst status extending to 2025. This not only corroborates the successful translation from basic research to clinical therapy but also underscores that the development of RNA-targeted nucleic acid drugs represents the most active frontier in the field (24). Collectively, this keyword burst map faithfully delineates the classic trajectory of translational medicine, progressing from fundamental mechanistic elucidation and animal model validation to eventual clinical drug discovery.

### 4.5 Semantic architecture revealed by topic modeling

Topic modeling based on NLP provides insights that extend beyond traditional bibliometric indicators. Conventional analyses identify surface-level patterns such as publication counts, keyword frequencies, or citation relationships, but they cannot fully capture the deeper semantic structure that links concepts across studies. In contrast, LDA produces data-driven topic clusters derived directly from the linguistic content of titles and abstracts. This approach uncovers the implicit thematic architecture of the field and reveals how mechanistic, pathological, and translational concepts co-occur in the research discourse (25).

Using LDA on the 620 SCI-E publications, we extracted six coherent topics that mapped onto three major axes: molecular mechanisms, disease-specific pathology, and translational frameworks (Figure 8). These topics highlight underlying conceptual connections that are not apparent from keyword co-occurrence alone. The results show that alternative splicing research has moved beyond isolated molecular descriptions toward a framework that integrates mechanistic regulation with disease modeling and therapeutic development. This deeper structure indicates that the field is becoming more conceptually unified and increasingly oriented toward clinically actionable splicing interventions.

Topics clustered around molecular mechanisms underscore the central importance of splice-site regulation and mutation-driven dysregulation, confirming that mechanistic precision remains the foundation of therapeutic innovation in neurodegenerative diseases. Alternative splicing is essential for normal neuronal function, and disruption of core splice-site selection or exon usage contributes directly to pathogenesis across disorders, as highlighted in recent high-impact reviews of splicing defects in neurodegeneration (14). The presence of distinct disease-focused clusters for ALS/FTD, Alzheimer’s disease, and SMA shows that splicing research is increasingly shaped by pathological heterogeneity rather than a single disease model. In ALS and FTD, loss of RNA-binding proteins such as TDP-43 leads to widespread mis-splicing and cryptic exon inclusion that alters neuronal gene expression and contributes to degeneration. For Alzheimer’s disease, splicing alterations linked to risk variants and regulatory imbalance have been associated with pathological isoforms of key genes, indicating that dysregulated splicing contributes to disease mechanisms beyond classical amyloid and tau pathology (21). The SMA-related topic, dominated by SMN-focused terminology, reflects the field’s most mature translational trajectory and aligns with the clinical success of splice-correcting therapies. Correction of *SMN2* exon 7 splicing increases functional SMN protein and improves clinical outcomes in patients, a strategy that has been validated in multiple high-impact preclinical and clinical studies (26).

The translational methodology topics indicate a clear shift toward platform-level development. Their focus on therapeutic design, target selection, and omics-based biomarkers shows that alternative-splicing therapeutics are moving from conceptual frameworks to practical implementation (27). Clinically validated examples support this transition (28). Nusinersen, the first splice-modifying therapy, improves *SMN2* exon inclusion and has transformed the treatment of SMA (29). Small-molecule modulators such as risdiplam further demonstrate that systemic splicing correction is feasible and effective (30). Preclinical studies extend this progress to broader neurodegenerative diseases. ASOs targeting cryptic exon activation in STMN2 or UNC13A restore protein expression and neuronal function in TDP-43 proteinopathy models (31). Early-phase clinical programs in ALS and Alzheimer’s disease are now testing related splicing-directed strategies (32).

### 4.6 Clinical trial paradigms: from supportive care to precision medicine

To capture the structural features of high-level clinical evidence, we applied CLARA clustering to clinical trials and randomized controlled trials retrieved from PubMed. The analysis clarifies the organization of clinical research in splicing-directed therapeutics. Two dominant clusters emerged (Figure 9A). Cluster 1 (blue) is defined by general methodological terms common in adult neurodegenerative research, indicating that current trial designs for this demographic remain broad and nonspecific. Cluster 2 (Yellow) is defined by pediatric and SMN-related terminology, showing that disease-focused splicing interventions are concentrated almost entirely in SMA. This distribution demonstrates that clinical translation is still heavily centered on SMA, a pattern driven by its clear genetic mechanism and the early clinical success of splice-correcting therapies. This suggests that meaningful expansion into adult neurodegenerative diseases will require the development of similarly rigorous, mechanism-based therapeutic platforms (33).

The subdivided SMA clusters reveal a bifurcated clinical trajectory (Figure 9B). Cluster 1 (green) features mechanism-based interventions, such as ASOs and SMN-targeted strategies. This indicates that splicing correction is now a central, standardized component of SMA therapy. Meanwhile, Cluster 2 (pink) highlights multidisciplinary supportive care, reflecting the ongoing need for respiratory, nutritional, and rehabilitative management. This divergence illustrates a dual-track clinical paradigm in SMA: the consolidation of a robust supportive care infrastructure alongside the rapid advancement and integration of groundbreaking molecularly targeted therapies.

The Supportive Era (pre-2016) reflects the dominance of symptomatic management in the absence of disease-modifying options (Figure 9C). The ASO Breakthrough Era (2017–2020) corresponds to the approval and post-marketing surveillance of nusinersen (34). The Precision and Screening Era (2021–2025) signals a strategic shift towards the presymptomatic application of these proven molecular interventions (35). Notably, the emergence of pyrimidines and RNA precursors (∼2025) coincides with the investigation of oral small-molecule modifiers (e.g., risdiplam), marking a transition towards systemic, scalable, and less invasive therapeutic platforms (36). Collectively, these trends delineate a maturing clinical landscape that has shifted from symptomatic management to targeted intrathecal interventions and, most recently, toward population-level precision strategies.

### 4.7 Funding structure evolution as a marker of translational maturity

In the ASO Breakthrough Era (2017–2020), for-profit funding surged to 7.89%—the highest proportion across any phase or era—reflecting commercial investment following regulatory approval. Notably, for-profit funding dropped to 0% in the subsequent Precision Era (2021–2025) (Figure 10). This withdrawal does not signal diminished industrial interest; rather, it reflects that direct product-focused research had matured, with subsequent studies on newborn screening, oral small-molecule optimization, and expansion to adult neurodegenerative diseases (ALS, Alzheimer’s disease) being sustained by non-profit foundations (e.g., Cure SMA, ALS Association) and public agencies.

These funding dynamics carry important implications for expanding splicing-targeted platforms beyond SMA. The successful SMA model suggests a staged funding architecture: early foundational support from disease-focused foundations, followed by public investment in preclinical validation, and finally for-profit capital for regulatory approval and market entry. For adult neurodegenerative diseases—where disease mechanisms are more heterogeneous and clinical trial costs are substantially higher—a similar coordinated funding strategy will likely be necessary, but with an even greater reliance on public–private partnerships and pre-competitive consortia.

### 4.8 Translational bottlenecks: from SMA success to mainstream NDDS

Integrating our keyword burst analysis (Figure 7C), LDA topic evolution (Figure 8A), and clinical trial clustering (Figures 9a,b), we delineate a three-phase translational trajectory and identify persistent bottlenecks that have hindered the expansion of splicing-targeted therapies beyond SMA.

Phase I (2000–2010) was dominated by basic splicing biology terms such as “pre-mRNA” and “alternative splicing.” Research was largely descriptive, with a scattered target landscape. A crucial exception was the elucidation of *SMN2* exon 7 skipping, which laid the mechanistic foundation for SMA.

Phase II (2011–2016) witnessed the emergence of “mice,” “survival motor neuron,” and “single nucleotide,” indicating the shift to in vivo validation. ASOs successfully corrected splicing in SMA transgenic mice, offering proof-of-concept. However, the model translation gap became evident: monogenic, early-onset SMA models differ fundamentally from complex, polygenic, late-onset AD/PD/sporadic ALS models. The latter often fail to reproduce progressive neuronal loss and age-dependent phenotypes, contributing to high attrition in human trials. Concurrently, delivery limitations emerged—ASOs require central administration, and intrathecal pharmacokinetic/safety data in large animals remained scarce.

Phase III (2017–present) is characterized by “nusinersen,” “drug discovery,” and “RNA” as bursting keywords. Clinical development has sharply diverged: SMA has established a dual-track paradigm (supportive care plus molecular intervention). In contrast, our clinical trial clustering (Figure 9A) shows that trials for adult NDDs (blue cluster) are enriched for generic methodological terms, lacking splicing-specific anchors. Three bottlenecks are now exposed:

(i) Model gap—Adult NDDs have weak genotype–phenotype correlations and no large animal models that can reliably predict clinical response.

(ii) Delivery constraints—The blood–brain barrier remains a significant obstacle. Intrathecal injection is unlikely to be scalable to the large adult NDD population; oral splicing modifiers (e.g., risdiplam) have limited brain exposure.

(iii) Biomarker deficit—SMA’s success depends on quantifiable target engagement (SMN protein) and stratification (*SMN2* copy number) biomarkers. Adult NDDs lack validated, non-invasive pharmacodynamic biomarkers for splicing correction in the brain.

Therefore, future strategies should prioritize: (a) next-generation BBB-penetrant delivery platforms; (b) parallel validation of pharmacodynamic biomarkers; and (c) development of predictive large-animal models with open-access translational databases.

## 5 Limitations

First, our primary data source was restricted to the SCI-E within the Web of Science Core Collection. Inevitably, this approach overlooks relevant publications indexed solely in other major databases, such as Scopus (37). This limitation might lead to a coverage bias, potentially underrepresenting research published in emerging regional journals or studies from fields like bioengineering and computational biology (38). As a result, our findings on research trends and collaborations mainly reflect the natural and medical sciences community covered by the WoSCC.

Secondly, the exclusion of non-English publications introduces a language bias. This is particularly relevant considering the substantial research output from non-English-speaking countries such as China, Germany, France, and Japan, some of which may be published in high-quality local journals (39). The low MCP ratio and centrality that we observed for countries such as China, for instance, could be partially influenced by this bias, as their domestic collaborations might be better documented in non-English sources.

The small number of PubMed-indexed clinical trials/RCTs (*n* = 10) reflects the current scarcity of high-evidence interventional studies targeting alternative splicing in neurodegenerative diseases beyond SMA, rather than a limitation of our search strategy. This finding itself is a key observation of our study, highlighting an urgent need for more rigorous clinical evaluation of splicing-modulating therapies in adult neurodegenerative populations.

Third, although LDA offered valuable insights into the latent thematic structure of the field, this methodology has inherent limitations. A different choice of k (e.g., 5, or 7 topics) could have resulted in a slightly different thematic granularity or clustering of concepts. Therefore, future research could utilize hierarchical LDA (hLDA) to uncover a topic hierarchy without pre-specifying the number of topics, or employ dynamic LDA to explore how the optimal number and nature of topics change over time, providing a more adaptive approach to thematic discovery (40, 41).

## 6 Conclusion

This study offers the first integrated bibliometric and NLP-driven analysis of global research on alternative splicing-based therapeutics for neurodegenerative diseases in the past 25 years (2000–2025). By integrating LDA topic modeling with traditional citation and collaboration network analyses, we identified latent thematic structures that conventional reviews cannot detect—showing how mechanistic, pathological, and translational concepts co-evolve throughout the literature. Three main findings emerge. First, the field has undergone a paradigm shift from descriptive molecular biology to clinically actionable splicing interventions. This shift has been accelerated by the regulatory approval of nusinersen in 2016 and the rise of small-molecule modulators such as risdiplam. Secondly, research leadership remains concentrated in the United States and Western Europe, with Harvard Medical School and University College London ranking at the forefront in terms of output and impact. Notably, the United States serves as a passive collaborative hub. Meanwhile, although China ranks second in output, it demonstrates the lowest international collaboration ratio (MCP% = 15.0%) and limited citation influence, which highlights the gaps in structural integration. Third, clinical translation remains highly skewed towards SMA. Trials in adult neurodegenerative diseases continue to be methodologically diffuse and mechanistically non-specific, highlighting the necessity for platform-level therapeutic strategies. Looking forward, the expansion of splicing-directed therapies will extend beyond SMA to ALS, Alzheimer’s disease, and frontotemporal dementia. This expansion will be driven by RNA-targeting platforms (ASOs, CRISPR) and systemic small molecules. Additionally, there will be an integration of early diagnostic tools (newborn screening, genetic testing) with presymptomatic intervention (42). Moreover, sustained progress will depend on expanding geographical participation, fortifying international collaboration, and cultivating cross-disciplinary platforms that link RNA biology, neurology, and data science (43, 44).

## Data Availability

The original contributions presented in this study are included in this article/Supplementary material, further inquiries can be directed to the corresponding authors.
